# Heterogeneous antimicrobial activity in broncho-alveolar aspirates from mechanically ventilated intensive care unit patients

**DOI:** 10.1080/21505594.2019.1682797

**Published:** 2019-10-29

**Authors:** Jolien Seinen, Willem Dieperink, Solomon A. Mekonnen, Paola Lisotto, Hermie J. M. Harmsen, Bart Hiemstra, Alewijn Ott, Daniel Schultz, Michael Lalk, Stefan Oswald, Sven Hammerschmidt, Anne Marie G. A. de Smet, Jan Maarten van Dijl

**Affiliations:** a Department of Medical Microbiology, University of Groningen, University Medical Center Groningen, Groningen, The Netherlands; b Department of Molecular Genetics and Infection Biology, Interfaculty Institute for Genetics and Functional Genomics, Center for Functional Genomics of Microbes, University of Greifswald, Greifswald, Germany; c Department of Critical Care, University of Groningen, University Medical Center Groningen, Groningen, The Netherlands; d Department Functional Genomics, Interfaculty Institute for Genetics and Functional Genomics, Center for Functional Genomics of Microbes, University Medicine of Greifswald, Greifswald, Germany; e Department of Medical Microbiology, Certe, Groningen, The Netherlands; f Institute of Biochemistry, University of Greifswald, Greifswald, Germany; g Department of Clinical Pharmacology, University Medicine of Greifswald, Greifswald, Germany

**Keywords:** *Streptococcus pneumoniae*, *Streptococcus anginosus*, *Staphylococcus aureus*, mechanical ventilation, sputum, antimicrobial activity

## Abstract

Pneumonia is an infection of the lungs, where the alveoli in the affected area are filled with pus and fluid. Although ventilated patients are at risk, not all ventilated patients develop pneumonia. This suggests that the sputum environment may possess antimicrobial activities. Despite the generally acknowledged importance of antimicrobial activity in protecting the human lung against infections, this has not been systematically assessed to date. Therefore, the objective of the present study was to measure antimicrobial activity in broncho-alveolar aspirate (‘sputum”) samples from patients in an intensive care unit (ICU) and to correlate the detected antimicrobial activity with antibiotic levels, the sputum microbiome, and the respective patients’ characteristics. To this end, clinical metadata and sputum were collected from 53 mechanically ventilated ICU patients. The antimicrobial activity of sputum samples was tested against *Streptococcus pneumoniae, Staphylococcus aureus* and *Streptococcus anginosus*. Here we show that sputa collected from different patients presented a high degree of variation in antimicrobial activity, which can be partially attributed to antibiotic therapy. The sputum microbiome, although potentially capable of producing antimicrobial agents, seemed to contribute in a minor way, if any, to the antimicrobial activity of sputum. Remarkably, despite its potentially protective effect, the level of antimicrobial activity in the investigated sputa correlated inversely with patient outcome, most likely because disease severity outweighed the beneficial antimicrobial activities.

## Introduction

In a healthy individual, the bacterial community of the lower respiratory tract mostly resembles that of the oral cavity [1,2]. However, bacteria are significantly less abundant in the lungs. This is related to effective clearing by a variety of mechanisms, including ciliated epithelium generating an “outward” flow of mucus, coughing [3,4] and immune defenses [5]. Nonetheless, disturbances in this well-balanced system may occur and ultimately lead to infectious diseases, pneumonia in particular [6].

Pneumonia is an infection of the lungs caused by bacteria, fungi or viruses, as stated by the World Health Organization (http://www.who.int/news-room/fact-sheets/detail/pneumonia), the Centers for Disease Control and Prevention (https://www.cdc.gov/pneumonia/) and others [7,8]. Literature differentiates between Community-Acquired Pneumonia (CAP) and Hospital-Associated Pneumonia (HAP). By definition, the onset of CAP takes place outside the hospital. However, since CAP may manifest itself shortly after the actual admission of patients in the hospital, cases of pneumonia occurring within 48 hours of hospital admission are usually defined as CAP. Accordingly, HAP is usually defined as a pneumonia occurring 48 hours or more after admission. Furthermore, the causative agents of CAP are oftentimes different and less resistant to antibiotics than those that cause HAP [9–11]. However, a precise distinction between CAP and HAP can be difficult. A particular risk factor is mechanical ventilation that may lead to so-called Ventilator-Associated Pneumonia (VAP). VAP usually arises 48 hours after endotracheal intubation [9,12], with a subdivision into early onset (within 4 days of hospital admission) and late onset VAP (after 4 days of hospital admission) [12]. Around 9–31% of the mechanically ventilated patients develop VAP depending on the clinical setting [13,14]. Main causative bacteria in VAP are *Staphylococcus aureus, Pseudomonas aeruginosa, Klebsiella pneumoniae, Escherichia coli, Enterobacter* species and (non-typeable; NTHi) *Haemophilus influenzae. Streptococcus pneumoniae* can also cause infection in VAP, but this is less common [15–19].

The alveolar sacs of the lung are filled with air in a healthy individual, but in patients with pneumonia the alveoli in the affected area are filled with pus and fluid (“sputum”) (World Health Organization, http://www.who.int/news-room/fact-sheets/detail/pneumonia). Sputum is an excessive amount of mucus with a composition that differs per individual and over time [20–23]. A main component is the glycoprotein mucin, but also DNA, filamentous actin, proteoglycans, biofilms, bacteria, antimicrobials and antibiotics may be present [20,24–27]. The accumulation of sputum in the lungs is a potential risk factor for pneumonia as it may represent an ecological niche for microorganisms. However, the fact that not all ventilated patients develop pneumonia suggests that the sputum environment may possess antimicrobial activities, which is a possibility that has not been systematically investigated to date. Accordingly, the objective of the present explorative study was to measure antimicrobial activity in broncho-alveolar aspirate samples (sputa) from patients in an intensive care unit (ICU) of the University Medical Center Groningen (UMCG), and to correlate the detected antimicrobial activity with antibiotic levels, the sputum microbiome and the respective patients’ characteristics.

## Material and methods

### Patients and sputum collection

**Figure 1.**
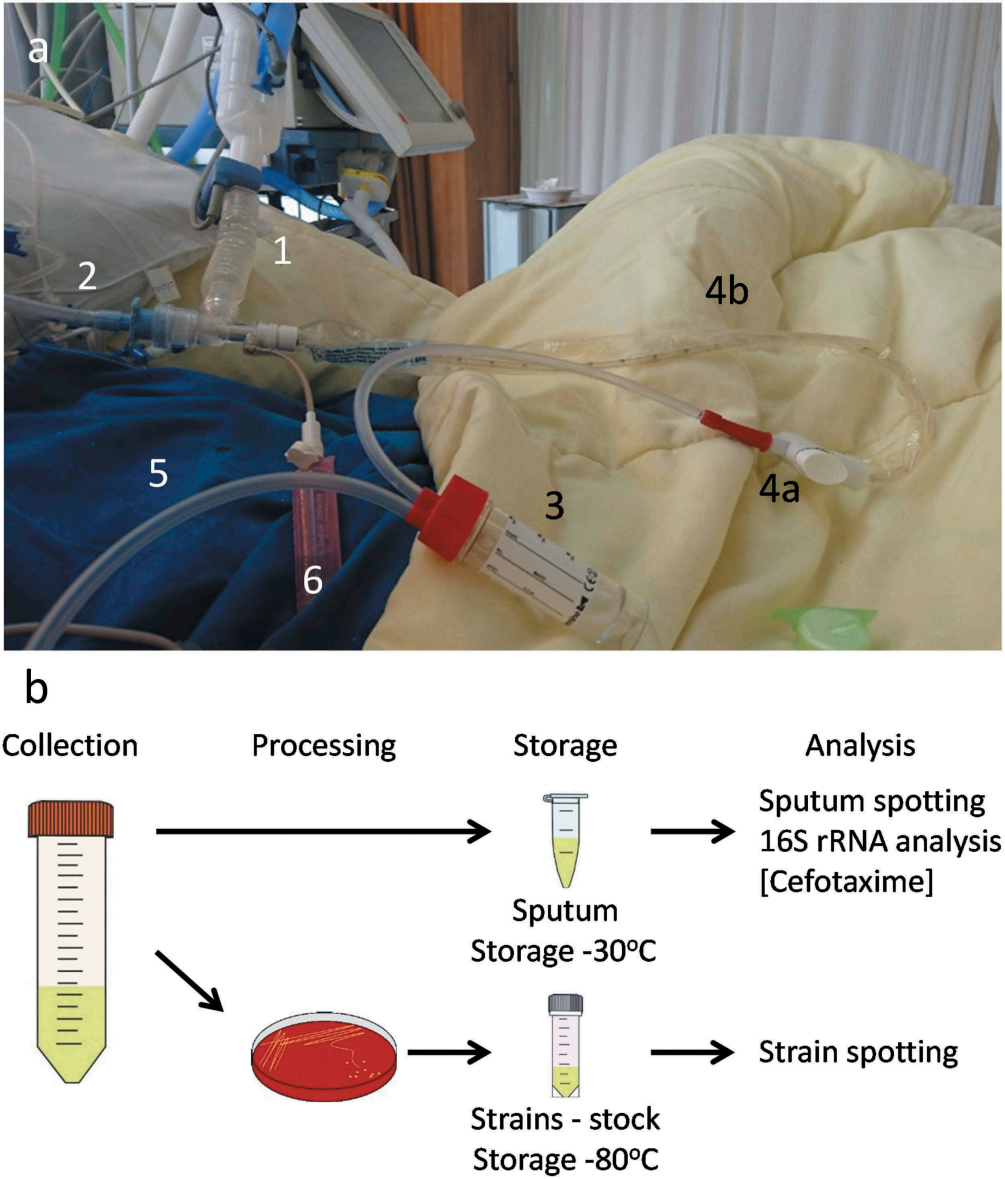
Schematic representation of sputum collection and sample processing, storage and analysis. (a) Image of sputum collection from a mechanically ventilated patient. A mechanical ventilation machine [1] supplies the intubated patient [2] with warm humidified air. Sputum is collected by attaching an external collection tube [3] to a tube (4a-b) that is connected to the intubation tube. The tube on the other end [5] of the collection tube is connected to a vacuum pump. The vacuum can be applied to this closed system by pressing a button (4a), and the tube for sputum collection (4b) is then inserted into the patient’s lungs. Some saline [6] can simultaneously be introduced into the lung to ease the sputum extraction. (b) Schematic representation of the workflow following sputum collection. The collected sputum is (i) processed for storage and further analyses (i.e. spotting on indicator bacteria, determination of residual antibiotics and 16S rRNA analysis), and (ii) plated for collection of microorganisms present in the sputum and subsequent assessment of the production of antimicrobial agents (i.e bacteriocins) by spotting on plates with indicator bacteria.

From February to August 2015, nurses collected sputum from mechanically ventilated patients (Figure 1) admitted to the department of Critical Care of the UMCG. Patient exclusion criteria were suspicion or diagnosis of tuberculosis, fungal or viral infections, immunodeficiency, administration of cytostatic agents, or positive end expiratory pressure >10 cm H_2_0. Diagnostic culturing of sputa was performed within 24 h after sampling according to the standard diagnostic routine at the UMCG. Sputum samples used for other analyses were initially stored at 4°C, and as soon as possible aliquoted and frozen at −20°C. Where possible, multiple samples from the same patient were collected and numbered chronologically. Initially, 58 patients were enrolled in this study. However, in the course of the study, it turned out that 1 patient was enrolled twice and 4 patients were excluded, either because of treatment with cytostatic agents, loss of the sputum sample, or inconsistency of recorded patient data. Ethical approval for this study was obtained from the Medical Ethical Committee of the UMCG (research project number 2014.309), which decided that informed consent was not necessary since all patients admitted to the UMCG are informed that their data and (diagnostic) waste materials can be used for scientific research. All patient data and samples were collected with adherence to the Helsinki Guidelines and processed anonymously.

### Collection of sputum-resident microorganisms

Before storing sputum samples at −20°C, they were streaked on 5% sheep blood agar (BA), chocolate agar (CHOC), and MacConkey agar no.3 (MCC3) plates (Mediaproducts BV, the Netherlands). Plates were incubated at 37°C with (BA, CHOC) or without (MCC3) 5% CO_2_ according to standard diagnostic routine. Single colonies were then picked and, after subculture, the respective microorganisms (bacteria and yeasts) were suspended in tryptic soy broth (TSB, Oxoid) with 11% glycerol and stored at −80°C.

### Bacterial strains and growth conditions

*S. pneumoniae* TIGR4 (serotype 4) [28] and *Streptococcus anginosus* 009–1 (clinical isolate from sputum sample 009–1, this study) were grown as standing cultures in M17 broth (Oxoid) with 0.5% glucose (GM17) at 37°C. *S. aureus* HG001 [29] was grown in TSB at 37°C under shaking conditions (250 rpm).

### Sputum spotting assay to measure antimicrobial activity

Large BA plates were prepared by pouring blood agar base no.2 (Oxoid) supplemented with sheep blood (Thermo Scientific; 5% final concentration) into Nunc™ Square BioAssay Dishes (245x245x25 mm, Thermo Scientific). The bacterial indicator strains *S. pneumoniae* TIGR4, *S. anginosus* 009–1 and *S. aureus* HG001 were grown overnight in GM17 or TSB, diluted in fresh medium and grown till an optical density at 600 nm (OD_600_) of ~0.25 (*S. pneumoniae* TIGR4 and *S. aureus* HG001) or ~0.14 (*S. anginosus* 009–1). 450 µl of culture was streaked evenly on a BA plate, using a cotton swab that was humidified with phosphate-buffered saline (PBS). Sputum samples were quickly thawed in a 37°C water bath, vortexed and aliquots of ~15 µl were spotted on BA plates with the respective indicator bacteria. These plates were then incubated overnight at 37°C and 5% CO_2_. Images were recorded with a G:Box (Syngene, Leusden, the Netherlands), and antimicrobial activity of sputum samples was assayed by measuring the size of growth inhibition zones with ImageJ [30].

### Cefotaxime quantification in sputum samples and Etest

Mechanically ventilated patients at the department of Critical Care of the UMCG are subject to selective decontamination of the digestive tract (SDD) if the duration of mechanical ventilation is expected to exceed 48 h and/or if the expected duration of the admission exceeds 78 h [31]. SDD is intended to prevent secondary colonization with Gram-negative bacteria, *S. aureus*, and yeasts through: (i) the application of non-absorbable antimicrobial agents (i.e. tobramycin, colistin, and amphotericin B) in the oropharynx and gastrointestinal tract, and (ii) preemptive treatment of possible infections with commensal respiratory tract bacteria through systemic administration of cephalosporins (especially cefotaxime) during the patient’s first four days in the ICU [31]. To determine cefotaxime concentrations in sputum, flash-frozen sputum aliquots of ~100 µl were cryofractured twice with a cryoPREP CP02 (Covaris) at maximum power using tissueTUBE TT1 Extra Think sample bags (Covaris). Sample extraction was subsequently performed by adding 1000 µl ice-cold 60% methanol and centrifugation (17,096 x g, 3 min, 4°C). Extracts were stored at −20°C until further analysis. To process the samples for high-performance liquid chromatography (HPLC) – mass spectrometry (MS) analyses, 100 µl deionized water was added to 200 µl of extract, followed by the addition of 25 µl 1% citric acid and 25 µl of the internal standard 4-hydroxychalcone (1 µg/ml; Sigma). Methanol was removed by vacuum evaporation (Scanvac) at 2000 rpm, for 15 min at room temperature. HPLC-MS/MS analyses were performed with an Agilent 1100 series HPLC system (Agilent Technologies, Waldbronn, Germany) coupled to an API4000 mass spectrometer (Sciex, Darmstadt, Germany) using an Atlantis® Silica HILIC column (3 µm 2.1 × 100 mm; Waters Corporation, Milford, MA) with isocratic elution (50% acetonitrile, 50% ammonium acetate (5 mM, pH 3.8)) at a flow rate of 200 µl/min. The lower limit quantification for cefotaxime in sputum extracts was 0.8 ng/ml. Because of the viscosity of sputum, the weight of each sample was measured and the cefotaxime concentration was calculated based on the assumption that sputum has an average density of one.

The minimal inhibitory concentration (MIC) of *S. pneumoniae* TIGR4, *S. anginosus* 009–1 and *S. aureus* HG001 for cefotaxime was determined with M.I.C.Evaluator strips (MA0111D, Oxoid) on Mueller Hinton II agar with 5% sheep blood (for streptococci) or regular Mueller Hinton agar (EUCAST; for *S. aureus*) (Mediaproducts BV, the Netherlands).

### Strain spotting assay to detect antimicrobial activity produced by sputum isolates

Bacteria and yeasts isolated from sputum samples with antimicrobial activity were grown overnight as standing cultures in Brain Heart Infusion (BHI) broth (Oxoid) or TSB at 37°C and 5% CO_2_. The overnight cultures were vortexed and aliquots of 2 µl were spotted on BA plates with indicator bacteria. In those cases where sputum isolates did not grow in BHI or TSB, the respective isolates were grown overnight on BA plates. Subsequently, colonies were suspended in PBS and aliquots of 2 µl were used for spotting on BA plates with indicator bacteria. Plates with indicator bacteria and spotted sputum isolates were incubated and analyzed as described above.

### Bacterial identification by MALDI-TOF MS

The *S. anginosus* sputum isolate from sample 009–1 was identified by MALDI-TOF MS as described previously [32]. In brief, the isolate was cultured on blood agar at 37°C and 5% CO_2_. Individual colonies were transferred in duplicate onto a stainless-steel MALDI target using a toothpick. Upon drying at room temperature, 1 μl of a matrix solution, composed of α-cyano-4-hydroxycinnamic acid in 50% acetonitrile/2.5% trifluoro-acetic acid (HCCA), was added to the first spot. For so-called on-target extraction, the second spot was overlaid with 1 μl of 70% formic acid prior to the addition of 1 μl HCCA matrix. The samples were then analyzed with a Bruker microflex MALDI-TOF MS system using the Biotyper 3.0 software (Bruker Daltonik, Bremen, Germany).

### 16S rRNA sequencing

Sputum aliquots of ~100 µl were used for total DNA extraction with the Zymo Quick DNA kit (Zymo Research, CA, USA). A liquid culture of *S. pneumoniae* TIGR4 served as a positive control, and the kit ingredients were used as a negative control. Polymerase chain reaction (PCR) amplification, PCR cleanup, MiSeq library preparation and sequencing with an Illumina MiSeq System (Illumina Inc. San Diego, USA) were performed as described by Heida et al. [33]. Briefly, sputum DNA was used to amplify the V3-V4 region of the 16S rRNA gene by PCR using modified 341F and 806R primers with a 6-nucleotide index sequence on the 806R primer, as published by Bartram et al. [34]. The resulting FASTQ files with Illumina paired-end reads were processed with PANDAseq, the taxonomy at phylum, family and genus levels was defined with the open source software package QIIME, and ARB was subsequently used to define isolates at the species level [33,35,36]. Samples with less than 1000 reads in total were excluded from further analysis. Heatmaps were generated by using R package version 3.3.3 and edited with Adobe Illustrator CC 2017.

The BAGEL4 webserver was used to determine the possible presence of genes encoding bacteriocins or ribosomally synthesized and post-translationally modified peptides (RiPPs) in genomes of interest [37]. These analyses were based on up to three randomly selected publicly available genome sequences of species identified by 16S rRNA sequencing.

### Statistical analyses

Statistical analyses, including Principal Component Analyses (PCA), t-tests, Mann-Whitney U tests, Pearson’s chi-squared tests and a Spearman test were performed with the Statistical Package for Social Science version 25 (SPSS, IBM). A p-value ≤ 0.05 was considered statistically significant.

## Results

### Cohort of mechanically ventilated ICU patients

To collect sputum samples, a study cohort of 53 patients was recruited from 58 eligible mechanically ventilated patients admitted to the Neuro ICU of the department of Critical Care of the UMCG. The baseline and clinical characteristics variables of the patients included in this study are summarized in Table 1. An overview of the antibiotics administered to the patients during ICU admission is presented in Supplemental Table S1. Of note, no information is available on antibiotics prescribed prior to admission to the ICU.

**Table 1.** Baseline characteristics and clinical variables of included ICU patients (n = 53).

Variables	Median [IQR] {range} or n (%)
Gender	
Male	32 (60.4)
Female	21 (39.6)
Age median (years)	58.0 [41.5–71.0] {19.0–85.0}
Hospital LOS (days)	18.6 [9.8–32.2] {0.8–75.5}
ICU LOS (days)	10.2 [5.5–19.8] {0.8–75.4}
Admission diagnosis	
Neurological	39 (73.6)
Respiratory	6 (11.3)
Medical	3 (5.7)
Cardiological	3 (5.7)
Gastroenterological	2 (3.8)
ICU outcome	
Hospital Transfer	37 (69.8)
Deceased	14 (26.4)
Nursing home	2 (3.8)
Mech. Vent. (hours)	134.0 [84.0–301.0] {13.0–1809.0}
COPD	2 (3.8)
Pneumonia	18 (34.0)
SAPS II	49.0 [37.0–57.5] {19.0–72.0}
APACHE IV^a^	78.0 [58.5–88.0] {29.0–126.0}
I.V. antibiotics	44 (83.0)
SDD topical antibiotics	42 (79.2)
Corticosteroids	16 (30.2)
Leukocytes	
Sample^§b^	12.3 [9.3–15.4] {4.4–30.0}
Lowest^§§^	8.0 [6.0–9.6] {2.1–17.7}
Highest^§§§^	18.3 [15.4–23.7] {8.1–53.2}
CRP	
Sample^§b^	68.0 [37.0–131.5] {1.8–319.0}
Lowest^§§^	5.5 [1.4–27.5] {0.3–264.0}
Highest^§§§^	133.0 [76.0–210.0] {16.0–465.0}

IQR, interquartile range; LOS, length of stay; ICU, intensive care unit; Mech. Vent., mechanical ventilation; COPD, Chronic Obstructive Pulmonary Disease; SAPS, Simplified Acute Physiology Score; APACHE, Acute Physiology and Chronic Health Evaluation; I.V., intravenous; SDD, selective decontamination of the digestive tract; CRP, C-reactive protein. ^§^Leukocytes/CRP measured in blood at the time of first sputum sample collection, ^§§^Lowest leukocytes/CRP measured in blood during ICU admission, ^§§§^Highest leukocytes/CRP measured in blood during ICU admission. ^a^Available for 50 patients; ^b^available for 49 patients.

### Antimicrobial activity in patient sputa

**Figure 2.**
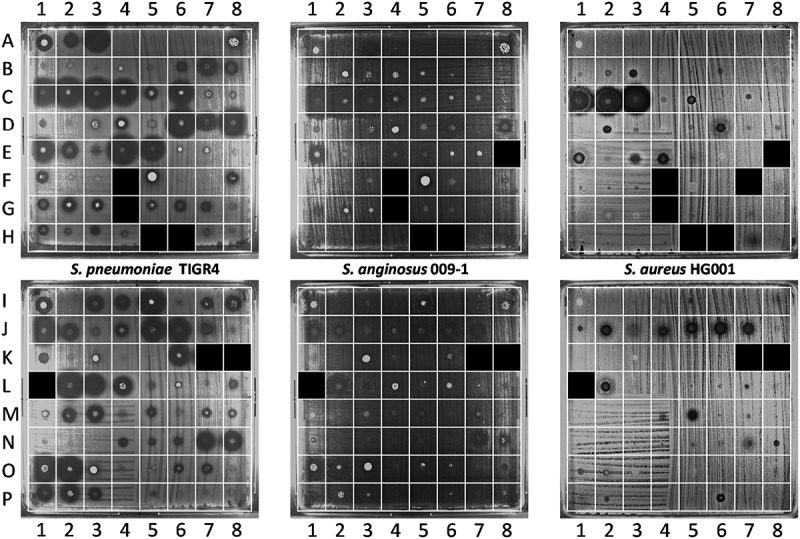
Detection of antimicrobial activity in sputa. 125 sputum samples from 56 different patients were spotted on blood agar plates with confluent lawns of the indicator bacteria *S. pneumoniae* TIGR4, *S. anginosus* 009–1 and *S. aureus* HG001. The antimicrobial activity in particular sputa is depicted by cleared zones of growth inhibition. Of note, this assay was performed before completion of the analysis of patient data. Since three patients were subsequently excluded from the study (see materials and methods), the results for the six respective sputum samples (G4, H5-6, K7-8 and L1) are covered in the image. One sputum sample (F4) could not be applied to the plates, because the sputum sample was too viscous for quantitative spotting; for two other samples the results are covered (E8, F7), because insufficient amounts of sputum were available to test them against all three species of indicator bacteria. The topography of sputum samples spotted onto lawns of the indicator bacteria is described in Supplemental Table S2.

Sputum samples were collected by aspiration during routine ICU patient care as represented in Figure 1. Subsequently, the collected sputa were tested for antimicrobial activity in a spotting assay, where aliquots were transferred to a plate with the indicator bacteria *S. pneumoniae* TIGR4, *S. anginosus* 009–1 or *S. aureus* HG001. Here the *S. anginosus* isolate 009–1 served as a sputum-resident control bacterium. Upon overnight incubation at 37°C, antimicrobial activity in particular sputum samples was evidenced by zones of growth inhibition of the indicator bacteria. The results are shown in Figure 2. Overall, large differences in the extent of growth inhibition were observed for different sputum samples from different patients (Table S2 and S3). Furthermore, the highest degree of “sputum sensitivity” was observed for *S. pneumoniae*, whereas *S. aureus* displayed the lowest sensitivity to the applied sputa. In extreme cases, major growth inhibition was observed for *S. pneumoniae*, while *S. aureus* was not inhibited at all (Figure 2; A2-3, B7, C6, D7-8, E2, E5, I3-5, K6, L3-4, O2, P1 and P3). Conversely, three sputa inhibited the growth of *S. aureus* without affecting *S. pneumoniae* (Figure 2; B3, D2, P6). The sensitivity of *S. anginosus* to the different sputa was largely comparable to that of *S. pneumoniae*, albeit that the analysis also showed several clear differences (Figure 2; A3, B6-7, C5-6, D7, E2, F1, I3-4, K6, L4, M2-3, and P1-3). Of note, 25 sputa displayed no detectable antimicrobial activity against the three indicator bacteria (Figure 2; A4-8, B1-2, B4-5, E6-7, F2-3, F6, I2, K2, K4-5, N2-3, O5, O8, P4, and P7-8). Together, these observations show that many of the sputa displayed significant antimicrobial activity, which may have originated from antimicrobial therapy of the respective patients (Supplemental Table S1), the “sputum microbiota”, or the patient’s innate immune defenses.

### Detection of cefotaxime in isolated sputa

**Figure 3.**
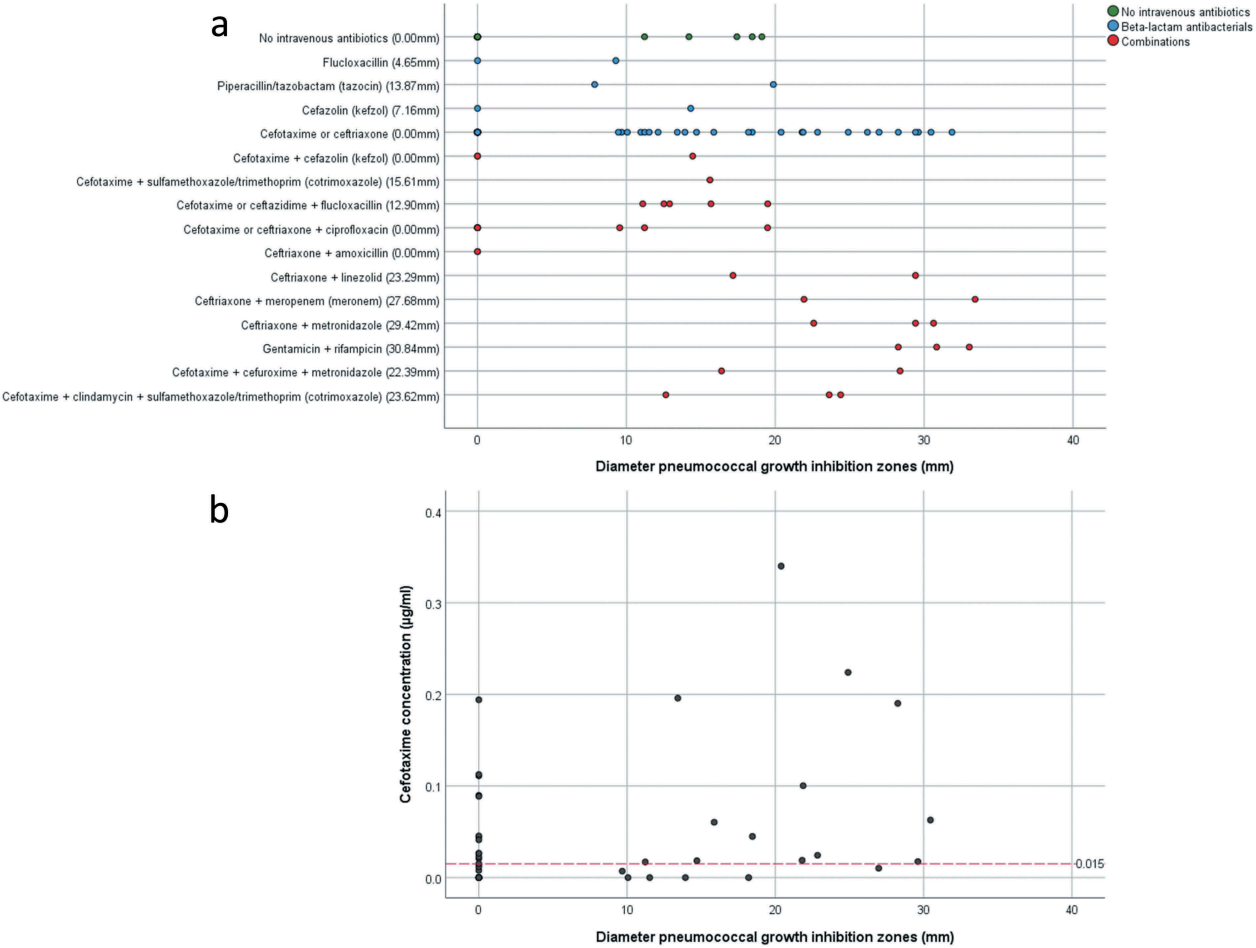
Pneumococcal growth inhibition related to administered antibiotics or actual cefotaxime concentrations in collected sputum samples. (a) Scatter plot of pneumococcal growth inhibition versus the intravenously administered antibiotics as detailed in Supplemental Table S1. The median inhibition is shown behind the (combination of) antibiotics on the Y-axis. (b) Scatter plot of pneumococcal growth inhibition versus the measured cefotaxime concentration in sputum. The red dashed line indicates the MIC of cefotaxime for *S. pneumoniae* TIGR4. Note that several samples with cefotaxime concentrations above the MIC for *S. pneumoniae* TIGR4 show no growth inhibition, whereas other samples with cefotaxime concentrations lower than the MIC for *S. pneumoniae* TIGR4 show strong pneumococcal growth inhibition. Importantly, cefotaxime was only measured in samples from patients who received cefotaxime as monotherapy during ICU admission.

Forty-four of the 53 included patients had received antibiotics intravenously, either to treat or prevent an infection. In particular, 33 patients were treated with cefotaxime; 25 of these patients received this antibiotic as monotherapy, whereas 3 patients were treated with cefotaxime in combination with ciprofloxacin and 5 patients with cefotaxime and another antibiotic. Eleven patients were treated with (combinations of) other antibiotics (Supplemental Table S1, Figure 3(a)). Since cefotaxime was most frequently used as part of the SDD-infection-prevention regimen, we examined the presence of this antibiotic in sputum by HPLC-MS/MS. Of note, cefotaxime was only measured in samples from those patients who received cefotaxime as a monotherapy, provided that sufficient sputum was available for this analysis. The results presented in Table S4 show that the measured cefotaxime concentrations in sputum samples ranged from zero to 0.340 µg/ml. In fifteen samples no cefotaxime was detectable. Remarkably, the measured cefotaxime concentration in sputa cannot be directly related to the pneumococcal growth inhibition zones observed upon sputum spotting, as shown by the scatter plot in Figure 3(b). A Spearman test confirmed this observation with a non-significant (p = 0.767) correlation coefficient of 0.052 (n = 35). Of note, samples without a quantifiable cefotaxime concentration and pneumococcal growth inhibition zone were excluded from the Spearman test (n = 11), to avoid a spurious overestimation of the correlation. Moreover, in particular sputum samples in which cefotaxime was detectable by HPLC-MS/MS, no growth inhibition of *S. pneumoniae* was observed (Table S4; Figure 2). This implies that at least part of the sputum-associated cefotaxime may be present in a state in which it cannot exert its antibiotic activity.

### Potential antimicrobial activity produced by sputum microbiota

**Figure 4.**
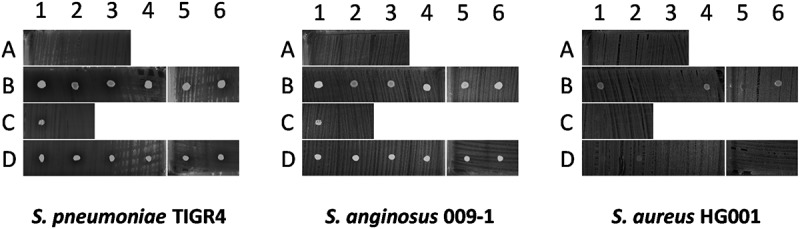
Antagonistic actions between bacterial sputum isolates and indicator bacteria. 14 microbial isolates from seven sputum samples of three different patients were spotted on blood agar with confluent lawns of the indicator bacteria *S. pneumoniae* TIGR4, *S. anginosus* 009–1 and *S. aureus* HG001. Note that none of the spotted isolates caused growth inhibition of the indicator bacteria, whereas *S. aureus* HG001 did inhibit growth of some of the spotted isolates. The topography of bacterial samples spotted onto lawns of the indicator bacteria is detailed in Supplemental Table S5.

Remarkably, in some cases the measured concentration of cefotaxime was below the MIC value of *S. pneumoniae* TIGR4 (0.015 µg/ml), while the respective sputa still inhibited growth (Table S4; Figures 2 and 3(b)). Moreover, four samples from two patients who did not receive antibiotics intravenously showed significant pneumococcal inhibition (Figure 2; I3-4 and P2-3). This was suggestive of antimicrobial activity produced by the sputum microbiota or the patient. To test the influence of the “sputum microbiome”, microbes isolated from sputa with antimicrobial activity were spotted onto BA plates with the indicator bacteria *S. pneumoniae* TIGR4, *S. anginosus* 009–1 or *S. aureus* HG001. Of note, only isolates from patients who had not been intravenously treated with antibiotics were selected for this analysis to avoid false-positive results. Upon overnight incubation at 37°C, plates were examined for possible growth inhibition. As shown in Figure 4, some of the spotted isolates (Table S5) exerted marginal growth inhibitory effects on *S. pneumoniae*, while zero growth inhibition of *S. anginosus* and *S. aureus* was observed. On the other hand, *S. aureus* HG001 exerted antimicrobial activity on several spotted sputum isolates and allowed only significant growth of four spotted isolates (Figure 4; B1, B4, B6, D2). This is consistent with the presence of an operon for production of the lantibiotic Gallidermin in the genome of *S. aureus* HG001 as identified with the BAGEL4 algorithm.

### Identification of sputum microbiomes

**Figure 5.**
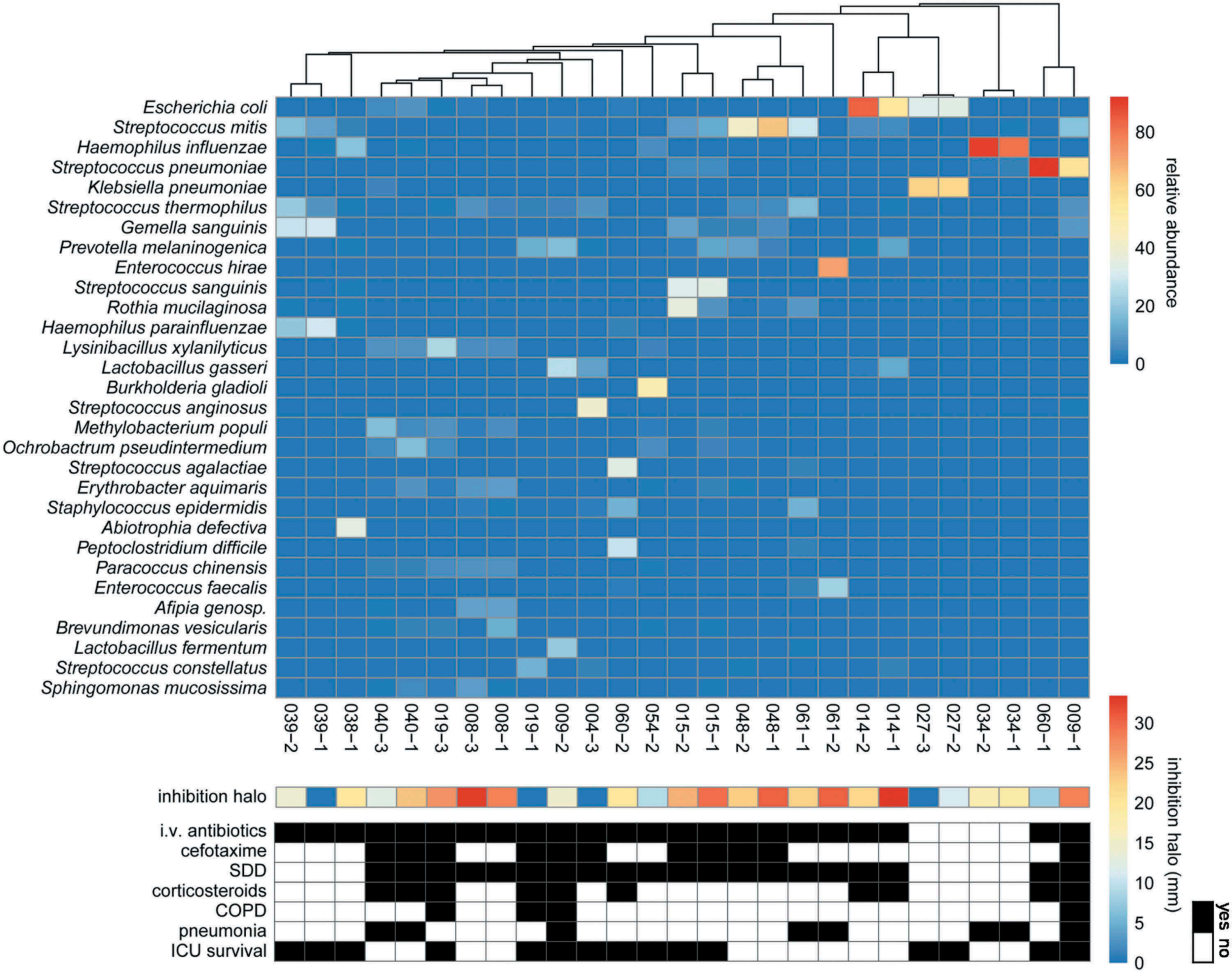
Heatmap of microbial abundance in sputum. The heatmap was generated based on a hierarchical clustering solution (Euclidean distance metric and average linkage) of the sputum microbiome samples (n = 27). Rows represent species identified by 16S rRNA sequencing, and columns represent individual sputum samples. The heatmap is sorted according to the dendogram analyses, based on the relative abundance of particular species. The color key for relative abundance of the different species is presented on the right of the heatmap. Positive and negative controls were performed, but the respective results are not presented in the heatmap. The panels below the heatmap present the detected antimicrobial activity in each sputum sample (in mm; color-coded in accordance with the key on the right), and selected patient characteristics relating to (antimicrobial) therapy in the ICU, lung diseases and ICU survival (black indicates “yes”). Importantly, information in the latter panel is shown per sample. An additional bar plot of the microbial abundance per sputum sample is shown in Supplemental Figure S1, and a heatmap of the microbial abundance in sputum samples related to the respective antimicrobial activity against the indicator strain *S. pneumoniae* TIGR4 is presented in Supplemental Figure S3.

To investigate whether the microbiomes of sputa with or without antimicrobial activity isolated from particular patients differ, we analyzed the microbial population in 33 sputa by sequencing the respective 16S rRNA genes. Of note, the sample selection was based on two criteria, namely: (i) sputa from a particular patient displayed at least in one case antimicrobial activity against *S. pneumoniae*, but this patient had not been treated intravenously with antibiotics; and (ii) sputa from patients with at least one sample being inhibitory to both *S. pneumoniae* and *S. aureus*. Six out of the 33 samples were excluded after 16S rRNA sequencing, because their total number of reads were found to be less than the threshold of 1000 reads. In the remaining 27 samples 635 species were identified. The heatmap of Figure 5 is based on the 30 bacterial species that showed on average the highest relative abundance in all samples. Overall, the sputum microbiome was found to be highly heterogeneous with *Streptococcus thermophilus, Staphylococcus epidermidis*, and *Streptococcus mitis* being the most frequently identified sputum-resident bacteria. These bacteria were detected in 27, 26 and 25 sputum samples, respectively. Importantly, 16S rRNA sequencing identified *S. anginosus* in sputum sample 009–1 with a low relative abundance, but the detection of this species is consistent with its isolation from the same sample as described above. In general, bacterial species that were detected by routine diagnostic culturing were also detected through 16S rRNA sequencing (Table S6), while yeasts and fungi remained undetected due to the specific primers used.

Altogether, the 16S rRNA analysis showed that samples from the same patient mostly cluster together (Figure 5). Only the two samples from patient 009 and the two samples from patient 060 were separated, suggesting that the sputum microbiome of these patients had changed in the period between the collection of the two samples (see also Figure S1 and Table S7). Figure 5 also summarizes patient data on the administration of antimicrobial therapy and corticosteroids, lung diseases and ICU survival, but these data reveal no significant differences. Lastly, Figure 5 presents the anti-pneumococcal activity of the sputum samples as inferred from the size of the inhibition halos in Figure 2. The combined data show that there is no correlation between the sputum microbiome and antimicrobial activity against *S. pneumoniae* TIGR4. This view is supported by PCA analysis at the species level that revealed no differentiating clusters of inhibiting or non-inhibiting samples (Figure S2). To assess whether the species identified by 16S rRNA sequencing have the potential to produce antimicrobial activities, a BAGEL4 analysis was performed. This revealed the presence of predicted bacteriocin genes in 13 out of the 27 identified species, where it should be noted that for 3 identified species no genome sequence is publicly available (Figure S3). Yet, this bioinformatic analysis did not uncover possible correlations between the predicted potential to produce bacteriocins and the actual antimicrobial activities measured in the investigated sputa (Figure S3). These findings imply that antimicrobial activity in the investigated sputa is most likely due to therapeutic interventions and host factors, whereas there is little, if any, antimicrobial activity contributed by the sputum microbiome.

### Relationships between patient characteristics and antimicrobial activity

Mann-Whitney U, t-tests and Chi square analyses were performed to assess possible relationships between the patient characteristics and the antimicrobial activity against *S. pneumoniae* TIGR4 in either the first sputum sample collected from the different patients, or the averaged antimicrobial activities against *S. pneumoniae* TIGR4 in all samples from particular patients (Table S8). Two baseline characteristics, the SAPS II and APACHE IV scores, were significantly higher among patients with a first sputum sample with antimicrobial activity (Table S8). Patients with antimicrobial activity in their sputa on average also had significantly higher SAPS II scores and within this group, significantly more patients were treated intravenously with antibiotics (Table S8). Further, both among patients with antimicrobial activity in the first collected sputum sample and patients with antimicrobial activity in one or more sputa, significantly more patients received antibiotics intravenously for clinical reasons (Table S8, “other antibiotics”). Although the lowest measured CRP levels were significantly higher among patients with antimicrobial activity in the averaged samples (Table S8), there was no overall difference in the CRP levels of patients with or without antimicrobial activity in their sputa.

### Patient characteristics that influence the patient’s outcome

To investigate the possible influence of different patient characteristics on patients’ outcome in the ICU, the baseline characteristics of patients with or without pneumonia, and patients who survived or passed away in the ICU, were compared (Table 2). Patients with pneumonia stayed longer in the ICU as compared to patients without pneumonia, though overall the difference was not statistically significant. In addition, patients with pneumonia were significantly longer mechanically ventilated than patients who did not develop pneumonia, and they had higher CRP levels during collection of the first sputum study sample (Table 2). Compared to surviving patients, the patients who passed away in the ICU were significantly older, had higher SAPS II and APACHE IV scores, and showed higher levels of leukocytes, both in the category lowest and highest measured during ICU admission. The non-survivors had a significantly shorter length of stay in the hospital (Table 2). Remarkably, statistical analysis of the collected data showed that antimicrobial activity in the first sputum sample is significantly higher for patients who did not survive in the ICU than for the ICU survivors. In a similar analysis, where the average pneumococcal inhibition was compared for sputum samples from patients who passed away and patients who survived the ICU, no significant difference was observed (Table 2).

**Table 2.** Comparison of patient characteristics and antimicrobial activity against *S. pneumoniae* TIGR4 in sputum samples in relation to pneumonia and ICU survival.

Variable	Pneumonia (n = 18)	No pneumonia (n = 35)	p-value	ICU survivors (n = 39)	Non ICU survivors (n = 14)	p-value
Female gender	4 (22.2)	17 (48.6)	0.063	13 (33.3)	8 (57.1)	0.118
Age (years)	58.5 [33.0–71.0]	56.0 [44.0–72.0]	0.749	55.0 [36.0–69.0]	65.0 [58.8–77.3]	0.048*
Hospital LOS (days)	21.9 [11.4–34.5]	16.7 [8.6–27.5]	0.523	19.8 [12.9–33.8]	11.0 [4.8–24.6]	0.023*
ICU LOS (days)	15.0 [7.9–22.7]	7.6 [4.9–15.1]	0.055	10.3 [6.0–20.1]	9.3 [4.8–15.5]	0.600
Admission diagnosis						
Neurological	12 (66.7)	27 (77.1)	0.895	29 (74.4)	10 (71.4)	0.906
Respiratory	3 (16.7)	3 (8.6)		4 (10.3)	2 (14.3)	
Medical	1 (5.6)	2 (5.7)		2 (5.1)	1 (7.1)	
Cardiological	1 (5.6)	2 (5.7)		2 (5.1)	1 (7.1)	
Gastroenterological	1 (5.6)	1 (2.9)		2 (5.1)	0 (0.0)	
ICU outcome						
Hospital transfer	13 (72.2)	24 (68.6)	0.586			
Deceased	5 (27.8)	9 (25.7)				
Nursing home	0 (0.0)	2 (5.7)				
Mech. Vent. (hours)	280.0 [133.3–423.5]	115.0 [76.0–229.0]	0.010*	126.0 [78.0–297.0]	192.5 [110.5–329.0]	0.348
COPD	1 (5.6)	1 (2.9)	0.625	2 (5.1)	0 (0.0)	0.388
Pneumonia				13 (33.3)	5 (35.7)	0.872
SAPS II	51.1 ± 9.3	46.5 ± 14.0	0.163 ^(L 0.041)^	44.6 ± 11.4	57.5 ± 11.6	0.001* ^(L 0.956)^
APACHE IV^a^	81.4 ± 19.1	71.5 ± 24.5	0.145 ^(L 0.086)^	67.9 ± 18.6	97.8 ± 21.3	<0.001* ^(L 0.587)^
I.V. antibiotics	15 (83.3)	29 (82.9)	0.965	34 (87.2)	10 (71.4)	0.178
Cephalosporins (with or without other antibiotics)	15 (83.3)	26 (74.3)	0.456	32 (82.1)	9 (64.3)	0.173
Other antibiotics (with or without cephalosporins)	7 (38.9)	9 (25.7)	0.322	12 (30.8)	4 (28.6)	0.878
Only β-lactam antibiotics	9 (50.0)	25 (71.4)	0.123	27 (69.2)	7 (50.0)	0.198
Other antibiotics, with or without β-lactam antibiotics	6 (33.3)	4 (11.4)	0.054	7 (17.9)	3 (21.4)	0.775
No antibiotics	3 (16.7)	6 (17.1)	0.965	5 (12.8)	4 (28.6)	0.178
SDD topical antibiotics	15 (83.3)	27 (77.1)	0.599	32 (82.1)	10 (71.4)	0.401
Corticosteroids	5 (27.8)	11 (31.4)	0.784	12 (30.8)	4 (28.6)	0.878
Leukocytes						
Sample^§b^	13.0 [11.2–17.1]	11.3 [9.3–15.0]	0.319	12.2 [8.7–15.2]	13.3 [10.5–20.1]	0.172
Lowest^§§^	8.1 [6.3–9.7]	7.7 [5.6–9.6]	0.493	7.2 [5.7–9.3]	9.5 [7.9–12.1]	0.004*
Highest^§§§^	20.1 [17.0–26.8]	18.0 [12.8–23.4]	0.229	17.9 [14.3–22.9]	21.4 [18.3–25.9]	0.030*
CRP						
Sample^§b^	112.0 [52.0–264.8]	52.0 [20.5–114.0]	0.016*	68.5 [34.8–135.3]	52.0 [49.0–128.0]	0.801
Lowest^§§^	12.4 [2.1–57.8]	4.4 [1.0–20.0]	0.260	5.5 [2.3–22.0]	6.4 [0.7–46.5]	0.635
Highest^§§§^	166.0 [88.5–278.8]	119.0 [70.0–191.0]	0.086	133.0 [54.0–191.0]	162.0 [86.5–246.5]	0.323
First sample inhibition halo (mm)	10.4 [0.0–20.3]	0.0 [0.0–19.1]	0.259	0.0 [0.0–15.6]	20.5 [0.0–28.6]	0.006*
Average inhibition halo (mm)	12.0 [2.8–19.7]	4.7 [0.0–19.5]	0.304	4.8 [0.0–15.6]	16.3 [0.0–26.9]	0.083

The average *S. pneumoniae* TIGR4 growth inhibition is based on one to four sputum samples, depending on how many sputum samples were obtained from each patient. ^§^Leukocytes/CRP measured in blood at the time of first sputum sample collection, ^§§^Lowest leukocytes/CRP measured in blood during ICU admission, ^§§§^Highest leukocytes/CRP measured in blood during ICU admission. Statistical analyses included the Pearson’s chi-squared test (n (%)), the Mann-Whitney U test (median [IQR]) and the t-test (mean ± standard deviation).

* p-values ≤0.05 are considered significant. ^L^Levene’s test significance. ^a^Available for 50 patients; ^b^available for 49 patients.

As it is possible that the association between antimicrobial activity in the first sputum sample and ICU non-survival could be due to confounding by age or disease severity (judged by the SAPS II or APACHE IV scores), a multiple linear regression analysis was performed, using the first sample inhibition halo diameter as the dependent and ICU non-survival as the explanatory variable. Without the addition of age and/or disease severity as confounders, linear regression again showed a significant correlation (p = 0.003), but including only the SAPS II or APACHE IV score in the regression model disproved the relationship (p = 0.075 with the SAPS II, and p = 0.343 with the APACHE IV score). These findings imply that disease severity acted as a confounder, as both higher SAPS II and APACHE IV scores are associated with antimicrobial activity in the first sputum sample, explaining the apparent association between non-survival and antimicrobial activity.

## Discussion

The objective of this study was to assess the presence of possible antimicrobial activity in the lungs of mechanically ventilated patients, and to correlate this activity with relevant patient characteristics. Despite the generally acknowledged importance of antimicrobial activity in protecting the human lung against infections, this has not been systematically assessed to date. Accordingly, it was not known how antimicrobial activity correlates with patient characteristics, the composition of the lung microbiome and, most importantly, patient outcome. To address these questions with minimal patient discomfort, we took advantage of the fact that the aspiration of sputum is part of the routine care provided to mechanically ventilated ICU patients. Normally, the collected sputa are discarded, unless they are used for routine diagnostic culturing to assess possible lung infection. Here we show that sputa collected from different patients show a high degree of variation in antimicrobial activity, which can partially be attributed to antibiotic therapy. The sputum microbiome, although potentially capable of producing bactericidal agents (bacteriocins), seems to contribute in a minor way, if any, to the antimicrobial activity of sputum. Remarkably, despite the fact that antimicrobial activity is potentially protective, the level of antimicrobial activity in the investigated sputa correlated inversely with patient outcome in terms of ICU survival. However, the latter correlation should be attributed to a confounding effect of disease severity.

Substantial differences in growth inhibition by sputa from different patients were observed for the three indicator strains used in our study. Interestingly, *S. pneumoniae* TIGR4 was more sensitive to the tested sputa than *S. anginosus* 009–1 or *S. aureus* HG001. As mentioned, *S. anginosus* 009–1 was isolated from one of the investigated sputa and, accordingly, this isolate is probably well-adapted for survival in sputum. In contrast, *S. pneumoniae* TIGR4 and *S. aureus* HG001 are laboratory strains, whose ancestors were clinical isolates from the blood of septic patients [28,29]. The difference in ‘sputum susceptibility‘ between these two laboratory strains is in line with the fact that *S. aureus* is, in general, more robust than *S. pneumoniae* when cultured *in vitro*. While we cannot exclude the possibility that this difference in sputum susceptibility relates to strain-specific features, it is tempting to hypothesize that the observed difference is clinically relevant, because the incidence of VAP caused by *S. aureus* is up to 26-fold higher than that of VAP caused by *S. pneumoniae* [17–19]. In this respect, it will be interesting to investigate in future studies whether such differences in sputum susceptibility are also evident for Gram-negative bacteria implicated in VAP, such as *E. coli, H. influenzae, P. aeruginosa* or *K. pneumoniae.*

What is the nature of the observed antimicrobial activity in sputa? More than 80% of the patients included in this study received antibiotics intravenously, cefotaxime in particular. Because cefotaxime was detectable in 31 of 46 investigated sputa, it seems likely that the antimicrobial activity of these sputa is related to the presence of cefotaxime or cefotaxime-derived desacetylcefotaxime [38]. Yet, 13 sputa with cefotaxime concentrations higher than the MIC for *S. pneumoniae* TIGR4 showed no or marginal growth inhibition, suggesting that the cefotaxime present in these sputa cannot exert its antimicrobial activity. This might be due, for example, to sputum components like mucin, as has previously been shown for colistin [39]. Conversely, in six sputa from patients treated intravenously with cefotaxime, this antibiotic was not detectable or present below the MIC for *S. pneumoniae* TIGR4, indicating that the antimicrobial activity in these sputa may not relate (exclusively) to antibiotic therapy. Thus, it seems that the latter sputa contained antimicrobial compounds produced by the sputum microbiome or the respective patient’s innate immune system. However, it should be noted that mechanically ventilated patients in our study group were subject to SDD and that some of the applied non-absorbable antimicrobial paste may have leaked into the sputum. Also, some patients received antibiotics other than cefotaxime (Figure 3(a)), and we cannot fully exclude the possibility that some cefotaxime was degraded in the time between the testing for antimicrobial activity and the mass spectrometric assessment of cefotaxime concentrations.

None of the 14 bacteria isolated from sputum displayed significant antimicrobial activity against the three indicator strains in the strain-spotting assay. On the other hand, 13 out of the 27 bacterial species from the sputum microbiome, as identified by 16S rRNA sequencing, do contain genes for known bacteriocins. Thus, although not experimentally demonstrated, a possible contribution of the sputum microbiome to the detected antimicrobial activity of particular sputa is conceivable. The latter view would be supported by the fact that BAGEL4 revealed the presence of genes for synthesis of the lantibiotic Gallidermin in *S. aureus* HG001, which matches well with the observation that *S. aureus* HG001 inhibited the growth of ten bacterial sputum isolates. Even so, there was no correlation detectable between the sputum microbiome, the potential of species identified in the sputum microbiome to produce bacteriocins, and the actual antimicrobial activity against *S. pneumoniae* TIGR4 as measured in the different sputa. In particular, this is evident for *S. thermophilus*, a bacterial species harboring many bacteriocin genes that was almost evenly detectable in sputa with high or low antimicrobial activity. Intriguingly, several of the bacteria identified by 16S rRNA sequencing are notorious pathogens, such as *K. pneumoniae, E. coli, S. pneumoniae, H. influenzae* and *S. agalactiae*. It is remarkable that these pathogens were not always detected by routine culturing. This suggests that their growth may have been suppressed by antimicrobial therapy or other microbial species present in the sample, or that they were simply not recognized in a mixed culture. Further, it is noteworthy that the 16S rRNA analysis did not detect *S. aureus* in patients with pneumonia, despite the fact that *S. aureus* is one of the most isolated species from patients with VAP [17–19]. The latter may relate to the relatively small sample size in our study.

Lastly, patients in our study cohort, who did not survive hospitalization in the ICU, were significantly older, displayed higher disease severity scores (SAPS II, APACHE IV) and had a shorter length of stay in the hospital compared to patients who did survive in the ICU. While the latter associations can be expected, the present observation that antimicrobial activity against *S. pneumoniae* TIGR4 in sputa appeared to be inversely associated to ICU survival was unexpected. However, this apparent association is confounded by the disease severity, as inferred from the fact that higher SAPS II and APACHE IV scores were associated with antimicrobial activity in the first sputum sample. Possibly this relates to an impaired barrier function of the lung epithelium due to the patient’s frailty, which could allow more influx of antibiotics into the alveoli. Conceivably, these patients may also have displayed higher inflammatory responses at the time of sputum sampling, leading to higher levels of bactericidal compounds (e.g. defensins and cationic antimicrobial peptides) in the respective sputa. Leukocyte levels in blood were indeed higher for non-survivors, both in the category lowest and highest measured during ICU admission.

Interestingly, it was recently proposed that HAP may relate to a dysbiosis of the lung microbiome, where low-abundant and highly diverse microorganisms would be rapidly replaced by a relatively high load of particular respiratory pathogens, a process which could be exacerbated by the administration of antibiotics [11]. However, we do not believe that this scenario is applicable to patients included in our present study, because we did not observe an association between the administration of antibiotics and the risk of pneumonia.

In conclusion, the combined observations presented in this study reveal that the sputa of mechanically ventilated ICU patients contain variable levels of antimicrobial compounds, including antibiotics such as cefotaxime. The sputa display differential inhibitory activities toward different pathogens as evidenced for *S. pneumoniae, S. anginosus* and *S. aureus*. Nevertheless, the level of sputum antimicrobial activity correlated inversely with patient´s survival in the ICU, most likely because disease severity outweighed the potentially beneficial antimicrobial activities.

## Supplementary material

Supplemental data for this article can be acccessed here.
