# Supplementary material for: Heterogeneous antimicrobial activity in broncho-alveolar aspirates from mechanically ventilated intensive care unit patients
Source: Virulence. 2019 Oct 29;10(1):879–91. doi: 10.1080/21505594.2019.1682797 (PMC6844299; doi:10.1080/21505594.2019.1682797)
Supplement: Supplemental Material [file kvir-10-01-1682797-s001.zip › Seinen et al 2019_Figs S1-3_Tables S2-6 S8_revised.docx]

**Supplemental Figures 1 – 3**

**Supplemental Tables S2 – S6 and S8**

**Heterogeneous antimicrobial activity in broncho-alveolar aspirates from mechanically ventilated Intensive Care Unit patients**

Jolien Seinen^1,2^, Willem Dieperink^3^, Solomon A. Mekonnen^1,4^, Paola Lisotto^1^, Hermie J.M. Harmsen^1^, Bart Hiemstra^3^, Alewijn Ott^1,5^, Daniel Schultz^6^, Michael Lalk^6^, Stefan Oswald^7^, Sven Hammerschmidt^2#^, Anne Marie G. A. de Smet^3^ and Jan Maarten van Dijl^1#^

^1^Department of Medical Microbiology, University of Groningen, University Medical Center Groningen, The Netherlands

^2^Department of Molecular Genetics and Infection Biology, Interfaculty Institute for Genetics and Functional Genomics, Center for Functional Genomics of Microbes, University of Greifswald, Germany

^3^Department of Critical Care, University of Groningen, University Medical Center Groningen, The Netherlands

^4^Department Functional Genomics, Interfaculty Institute for Genetics and Functional Genomics, Center for Functional Genomics of Microbes, University Medicine of Greifswald, Greifswald, Germany

^5^Department of Medical Microbiology, Certe, Groningen, The Netherlands

^6^Institute of Biochemistry, University of Greifswald, Greifswald, Germany

^7^Department of Clinical Pharmacology, University Medicine of Greifswald, Greifswald, Germany

**^#^Correspondence:**

Jan Maarten van Dijl, Department of Medical Microbiology, University of Groningen, University Medical Center Groningen, Hanzeplein 1, P.O. Box 30001, 9700 RB Groningen, the Netherlands, E-mail: j.m.van.dijl01@umcg.nl

Sven Hammerschmidt, Department of Molecular Genetics and Infection Biology, Interfaculty Institute for Genetics and Functional Genomics, Center for Functional Genomics of Microbes, University of Greifswald, Felix-Hausdorff-Straβe 8, 17487 Greifswald, Germany, E-mail: sven.hammerschmidt@uni-greifswald.de

**Running title:** Antimicrobial activity in ICU patient sputa

**Figure S1. Bar plot of microbial abundance in sputum samples.** Sputum samples are indicated on the X-axis, and the relative abundance of particular identified species is shown on the Y-axis. The top-30 most abundant species are shown individually and marked by color code. Other identified species are clustered in the “others” category, which is presented at the top of each bar. The relative abundance values are shown in Supplemental Table S7.

**Figure S2. Principal component analysis (PCA) of the microbial composition in 27 sputum samples.** The PCA analysis was generated based on the percentages of reads of species in each sample. Groups are divided into inhibitory and non-inhibitory samples as seen in the spotting assay. Positive and negative controls were performed, but the respective results are not included in the PCA. PC1 describes 14.9% of the variation, PC2 11.8% and PC3 8.6%.

**Figure S3**. **Heatmap of microbial abundance in sputum samples related to the respective antimicrobial activity against indicator strain *S. pneumoniae* TIGR4.** The heatmap was generated based on a hierarchical clustering solution (Euclidean distance metric and average linkage) of the sputum microbiome samples (n = 27). Rows represent species identified by 16S rRNA sequencing, and columns represent individual sputum samples. The heatmap is sorted according to the detected antimicrobial activity in each sputum sample (in mm) as shown in the panel below. Both color keys are presented on the right of the heatmap. Positive and negative controls were performed, but the respective results are not presented in the heatmap. The average number and {range} of bacteriocin genes predicted by inspection of three different genomes of a particular bacterial species with BAGEL4 is indicated behind the respective species name. A dash indicates that there is presently no publicly available genome sequence and an asterix indicates there was only one genome available.

**Table S2**. Topography of sputum samples spotted onto lawns of the indicator bacteria *S. pneumoniae* TIGR4, *S. anginosus* 009-1 and *S. aureus* HG001 as shown in Figure 2.

|  | 1 | 2 | 3 | 4 | 5 | 6 | 7 | 8 |
| --- | --- | --- | --- | --- | --- | --- | --- | --- |
| A | Optochin | 001-1 | 001-2 | 002-1 | 002-2 | 002-3 | 002-4 | 003-1 |
| B | 004-1 | 004-2 | 004-3 | 005-1 | 005-2 | 005-3 | 006-1 | 007-1 |
| C | 008-1 | 008-2 | 008-3 | 009-1 | 009-2 | 009-3 | 010-1 | 010-2 |
| D | 011-1 | 011-2 | 013-1 | 013-2 | 013-3 | 014-1 | 014-2 | 015-1 |
| E | 015-2 | 018-1 | 019-1 | 019-2 | 019-3 | 020-1 | 020-2 | 020-3 |
| F | 020-4 | 021-1 | 021-2 | 021-3 | 023-1 | 023-2 | 023-3 | 024-1 |
| G | 024-2 | 024-3 | 024-4 |  | 026-1 | 026-2 | 026-3 | 027-1 |
| H | 027-2 | 027-3 | 028-1 | 028-2 |  |  | 032-1 | 032-2 |
|  |  |  |  |  |  |  |  |  |
| I | Optochin | 032-3 | 034-1 | 034-2 | 035-1 | 035-2 | 036-1 | 037-1 |
| J | 037-2 | 038-1 | 039-1 | 039-2 | 040-1 | 040-2 | 040-3 | 042-1 |
| K | 043-1 | 044-1 | 045-1 | 045-2 | 045-3 | 046-1 |  |  |
| L |  | 048-1 | 048-2 | 049-1 | 049-2 | 050-1 | 050-2 | 052-1 |
| M | 052-2 | 053-1 | 053-2 | 054-1 | 054-2 | 055-1 | 055-2 | 055-3 |
| N | 058-1 | 058-2 | 058-3 | 059-1 | 059-2 | 060-1 | 060-2 | 061-1 |
| O | 061-2 | 061-3 | 062-1 | 063-1 | 063-2 | 064-1 | 064-2 | 064-3 |
| P | 064-4 | 065-1 | 065-2 | 065-3 | 065-4 | 067-1 | 067-2 | 069-1 |

Numbers correspond with patient samples. Empty cells represent samples from patients who were excluded from our study. Further, sputum sample 021-3 was highly viscous and could therefore not be transferred quantitatively with a pipet onto lawns of indicator bacteria. The volume of sputum sample 020-3 was insufficient for testing on all three indicator bacteria and was, therefore, only tested against *S. pneumoniae* TIGR4. For the same reason, sputum sample 023-3 was only tested against *S. pneumoniae* TIGR4 and *S. anginosus* 009-1.

**Table S3**. Diameters of growth inhibition zones due to spotting of sputum samples onto a lawn of *S. pneumoniae* TIGR4 as shown in Figure 2.

| Patient | Sample 1 (mm) | Sample 2 (mm) | Sample 3 (mm) | Sample 4 (mm) | Average (mm) |
| --- | --- | --- | --- | --- | --- |
| 001 | 16.39 | 28.38 |  |  | 22.38 |
| 002 | 0.00 | 0.00 | 0.00 | 0.00 | 0.00 |
| 003 | 0.00 |  |  |  | 0.00 |
| 004 | 0.00 | 0.00 | 0.00 |  | 0.00 |
| 005 | 0.00 | 0.00 | 11.22 |  | 3.74 |
| 006 | 21.80 |  |  |  | 21.80 |
| 007 | 15.61 |  |  |  | 15.61 |
| 008 | 28.26 | 30.84 | 33.03 |  | 30.71 |
| 009 | 28.26 | 14.71 | 26.19 |  | 23.05 |
| 010 | 0.00 | 0.00 |  |  | 0.00 |
| 011 | 0.00 | 0.00 |  |  | 0.00 |
| 013 | 0.00 | 14.45 | 0.00 |  | 4.82 |
| 014 | 33.42 | 21.93 |  |  | 27.67 |
| 015 | 29.61 | 24.90 |  |  | 27.25 |
| 018 | 19.48 |  |  |  | 19.48 |
| 019 | 0.00 | 31.87 | 26.97 |  | 19.61 |
| 020 | 0.00 | 0.00 | 0.00 | 13.93 | 3.48 |
| 021 | 0.00 | 0.00 |  |  | 0.00 |
| 023 | 0.00 | 0.00 | 0.00 |  | 0.00 |
| 024 | 11.10 | 12.52 | 15.68 | 12.90 | 13.05 |
| 026 | 9.68 | 12.13 | 10.97 |  | 10.92 |
| 027 | 0.00 | 11.22 | 0.00 |  | 3.74 |
| 028 | 10.06 | 0.00 |  |  | 5.03 |
| 032 | 0.00 | 0.00 | 0.00 |  | 0.00 |
| 034 | 18.45 | 17.42 |  |  | 17.94 |
| 035 | 29.42 | 0.00 |  |  | 14.71 |
| 036 | 13.42 |  |  |  | 13.42 |
| 037 | 17.16 | 29.42 |  |  | 23.29 |
| 038 | 19.49 |  |  |  | 19.49 |
| 039 | 0.00 | 14.32 |  |  | 7.16 |
| 040 | 23.62 | 24.39 | 12.65 |  | 20.22 |
| 042 | 0.00 |  |  |  | 0.00 |
| 043 | 11.23 |  |  |  | 11.23 |
| 044 | 0.00 |  |  |  | 0.00 |
| 045 | 0.00 | 0.00 | 0.00 |  | 0.00 |
| 046 | 21.87 |  |  |  | 21.87 |
| 048 | 30.46 | 22.84 |  |  | 26.65 |
| 049 | 20.39 | 0.00 |  |  | 10.20 |
| 050 | 0.00 | 0.00 |  |  | 0.00 |
| 052 | 0.00 | 0.00 |  |  | 0.00 |
| 053 | 15.87 | 18.45 |  |  | 17.16 |
| 054 | 0.00 | 9.29 |  |  | 4.65 |
| 055 | 0.00 | 0.00 | 0.00 |  | 0.00 |
| 058 | 0.00 | 0.00 | 0.00 |  | 0.00 |
| 059 | 9.55 | 0.00 |  |  | 4.78 |
| 060 | 7.87 | 19.87 |  |  | 13.87 |
| 061 | 22.58 | 30.63 | 29.42 |  | 27.55 |
| 062 | 0.00 |  |  |  | 0.00 |
| 063 | 0.00 | 0.00 |  |  | 0.00 |
| 064 | 9.46 | 11.53 | 0.00 | 18.20 | 9.80 |
| 065 | 19.10 | 14.20 | 0.00 | 0.00 | 8.32 |
| 067 | 0.00 | 0.00 |  |  | 0.00 |
| 069 | 0.00 |  |  |  | 0.00 |

Diameters of growth inhibition zones were determined by image analysis with ImageJ.

**Table S4.** Cefotaxime concentrations in patient sputa.

| Patient | Sample | [cefotaxime] in µg/ml |  | Patient | Sample | [cefotaxime] in µg/ml |
| --- | --- | --- | --- | --- | --- | --- |
| 003 | 003-1 | 0.111 |  | **032** | 032-1 | 0.045 |
| 004 | 004-1 | **0.008*** |  |  | 032-3 | **ND**** |
|  | 004-2 | **0.014*** |  | **036** | 036-1 | 0.196 |
|  | 004-3 | 0.044 |  | **042** | 042-1 | **ND**** |
| 006 | 006-1 | 0.019 |  | **043** | 043-1 | 0.017 |
| 009 | 009-1 | 0.190 |  | **044** | 044-1 | **ND**** |
|  | 009-2 | 0.018 |  | **045** | 045-1 | **ND**** |
| 010 | 010-1 | 0.194 |  |  | 045-2 | **ND**** |
|  | 010-2 | 0.113 |  |  | 045-3 | **ND**** |
| 011 | 011-1 | **ND**** |  | **046** | 046-1 | 0.100 |
|  | 011-2 | 0.021 |  | **048** | 048-1 | 0.063 |
| 015 | 015-1 | 0.017 |  |  | 048-2 | 0.024 |
|  | 015-2 | 0.224 |  | **049** | 049-1 | 0.340 |
| 019 | 019-1 | **ND**** |  |  | 049-2 | 0.015 |
|  | 019-3 | **0.010*** |  | **052** | 052-1 | 0.089 |
| 020 | 020-1 | **0.012*** |  | **053** | 053-1 | 0.060 |
|  | 020-2 | 0.026 |  |  | 053-2 | 0.045 |
|  | 020-4 | **ND**** |  | **055** | 055-1 | 0.023 |
| 023 | 023-1 | 0.090 |  |  | 055-2 | 0.041 |
|  | 023-2 | **ND**** |  |  | 055-3 | 0.027 |
| 026 | 026-1 | **0.007*** |  | **064** | 064-2 | **ND**** |
| 028 | 028-1 | **ND**** |  |  | 064-3 | **ND**** |
|  | 028-2 | **ND**** |  |  | 064-4 | **ND**** |

*Concentration of cefotaxime below the *S. pneumoniae* TIGR4 MIC of 0.015 µg/ml. **ND, no cefotaxime detected.

**Table S5**. Topography of bacterial samples spotted onto lawns of the indicator bacteria *S. pneumoniae* TIGR4, *S. anginosus* 009-1 and *S. aureus* HG001 in the strain spotting assay as shown in Figure 4.

|  | 1 | 2 | 3 | 4 | 5 | 6 |
| --- | --- | --- | --- | --- | --- | --- |
| A | BHI* | TSB* | PBS* |  |  |  |
| B | 027-1 gn1 | 027-1 gn2 | 027-2 gn1 | 027-2 gn2 | 027-3 gn1 | 027-3 gn2 |
| C | 034-1 w1 | 034-1 w2 ** |  |  |  |  |
| D | 065-1 w | 065-1 flat | 065-2 w | 065-4 yeast 1 | 065-4 yeast 2 | 065-4 white |

* Negative controls; ** Bacterial cells from a BA plate resuspended in PBS.

**Table S6.** Comparison of results from routine diagnostic culturing of patient sputa and the top-3 most abundant bacterial species identified by 16S rRNA sequence analysis of sputa from the same patient as investigated in the present study.

| Patient | Diagnostic culturing | Sample | 16S:  Total number of reads | 16S:  Percentage |
| --- | --- | --- | --- | --- |
| 004 | - | 004-3 | 23054 | *S. anginosus* (41)  *C.* *gingivalis* (14)  *L. gasseri* (11) |
| 008 | No growth | 008-1 (*) | 1514 | *B. vesicularis* (13)  *Afipia* genosp. (11)  *E. aquimaris* (10) |
|  |  | 008-3 (*) | 1461 | *Afipia* genosp. (11)  *S. koreensis* (11)  *S. mucosissima* (10) |
| 009 | Aerobic Gram-positive mixed flora | 009-1 | 24239 | *S. pneumoniae* (57)  *S. mitis* (18)  *G. sanguinis* (8) |
|  |  | 009-2 | 24103 | *L. gasseri* (26)  *L. fermentum* (21)  *P. melaninogenica* (17) |
| 014 | *E. coli*  Yeast  *A. fumigatus* | 014-1 | 24591 | *E. coli* (54)  *L. gasseri* (13)  *P. melaninogenica* (12) |
|  |  | 014-2 | 22448 | *E. coli* (85)  *S. mitis* (6)  L. gasseri (2) |
| 015 | - | 015-1 (*) | 2593 | *S. sanguinis* (34)  *S. mitis* (13)  *P. melaninogenica* (12) |
|  |  | 015-2 | 23781 | *R mucilaginosa* (37)  *S. sanguinis* (33)  *G. sanguinis* (11) |
| 019 | No growth | 019-1 (*) | 7538 | *S. constellatus* (14)  *P. melaninogenica* (13)  *C. concisus* (10) |
|  |  | 019-3 (*) | 3634 | *L. xylanilyticus* (24)  *S. thermotolerans* (15)  *M. populi* (7) |
| 027 | *E. coli* | 027-2 | 18304 | *K. pneumoniae* (60)  *E. coli* (35)  *K. oxytoca* (4) |
|  |  | 027-3 | 21692 | *K. pneumoniae* (62)  *E. coli* (33)  *K. oxytoca* (4) |
| 034 | Aerobic Gram-positive mixed flora | 034-1 | 18627 | *H. influenzae* (81)  *N. meningitidis* (4)  *S. pneumoniae* (3) |
|  |  | 034-2 | 22635 | *H. influenzae* (90)  *N. meningitidis* (4)  *S. pneumoniae* (3) |
| 038 | Throat flora | 038-1 | 23041 | *A. defectiva* (36)  *H. influenzae* (18)  *P. salivae* (15) |
| 039 | Throat flora | 039-1 | 24694 | *H. parainfluenzae* (30)  *G. sanguinis* (30)  *S. mitis* (11) |
|  |  | 039-2 | 21353 | *G. sanguinis* (28)  *S. thermophilus* (21)  *H. parainfluenzae* (20) |
| 040 | Yeast | 040-1 (*) | 1015 | *O. pseudintermedium* (17)  *E. aquimaris* (8)  *E. coli* (8) |
|  |  | 040-2 (*) | 451 | No reliable identifications |
|  |  | 040-3 (*) | 2454 | *M. populi* (17)  *D. piger* (10)  *P. graminis* (8) |
| 048 | Throat flora | 048-1 | 21289 | *S. mitis* (65)  *G. sanguinis* (6)  *S. thermophilus* (5) |
|  |  | 048-2 (*) | 14581 | *S. mitis* (42)  *P. melaninogenica* (11)  *A. para-adiacens* (6) |
| 054 | - | 054-2 (*) | 8400 | *B. gladioli* (49)  *P. histicola* (8)  *H. influenzae* (6) |
| 060 | No growth | 060-1 | 24537 | *S. pneumoniae* (92)  *H. parahaemolyticus* (7)  *S. mitis* (1) |
|  |  | 060-2 | 17440 | *S. agalactiae* (35)  *P. difficile* (28)  *S. epidermidis* (14) |
| 061 | No growth | 061-1 (*) | 7545 | *S. mitis* (29)  *S. thermophilus* (17)  *S. epidermidis* (15) |
|  |  | 061-2 (*) | 11813 | *E. hirae* (71)  *E. faecalis* (23)  *E. durans* (2) |
|  |  | 061-3 (*) | 906 | No reliable identifications |
| 065 | Yeast | 065-1 (*) | 237 | No reliable identifications |
|  |  | 065-2 (*) | 255 | No reliable identifications |
|  |  | 065-3 (*) | 470 | No reliable identifications |
|  |  | 065-4 (*) | 386 | No reliable identifications |
| Negative control –  Buffers used for DNA isolation and PCR |  | (*) | 326 | *E. coli* (20)  *E. aquimaris* (12)  *Afipia* genosp. (8) |
| Positive control –  *S. pneumoniae* TIGR4 culture |  |  | 20268 | *S. pneumoniae* (96)  *E. coli* (1)  *P. bivia* (1) |

Note that for most patients, diagnostic culturing was performed only once. In case culturing was performed more than once, the results of culturing on the date closest to the collection date of sputum sampling for the present study is presented. ‘-‘ means that no diagnostic culturing of sputum was done, ‘No growth’ indicates that no microbial growth was detectable by diagnostic culturing. Study sputum samples marked with an (*) yield upon 16S rRNA sequencing >30% of reads relating to commonly encountered contaminants.

**Table S8**. Comparison of patient characteristics in relation to antipneumococcal activity in the first samples or the average antipneumococcal activity.

| Variable | No inhibition  sample 1  (n=26) | Inhibition  sample 1  (n=27) | p-value | No inhibition  average  (n=19) | Inhibition  average  (n=34) | p-value |
| --- | --- | --- | --- | --- | --- | --- |
| Female gender | 10 (38.5) | 11 (40.7) | 0.865 | 9 (47.4) | 12 (35.3) | 0.389 |
| Age (years) | 53.5 [24.0 – 70.5] | 59.0 [51.0 – 74.0] | 0.162 | 50.0 [24.0 – 72.0] | 59.0 [51.0 – 70.3] | 0.119 |
| Hospital LOS (days) | 17.8 [9.8 – 33.5] | 18.6 [9.5 – 31.9] | 1.000 | 15.8 [8.6 – 24.4] | 19.0 [10.3 – 34.5] | 0.504 |
| ICU LOS (days) | 11.0 [5.9 – 17.0] | 8.5 [5.0 – 21.5] | 0.943 | 11.7 [4.9 – 15.1] | 9.1 [5.7 – 22.6] | 0.809 |
| Admission diagnosis |  |  |  |  |  |  |
| Neurological | 21 (80.8) | 18 (66.7) | 0.578 | 17 (89.5) | 22 (64.7) | 0.239 |
| Respiratory | 3 (11.5) | 3 (11.1) |  | 2 (10.5) | 4 (11.8) |  |
| Medical | 1 (3.8) | 2 (7.4) |  | 0 (0.0) | 3 (8.8) |  |
| Cardiological | 1 (3.8) | 2 (7.4) |  | 0 (0.0) | 3 (8.8) |  |
| Gastroenterological | 0 (0.0) | 2 (7.4) |  | 0 (0.0) | 2 (5.9) |  |
| ICU outcome |  |  |  |  |  |  |
| Hospital transfer | 20 (76.9) | 17 (63.0) | 0.091 | 14 (73.7) | 23 (67.6) | 0.756 |
| Deceased | 4 (15.4) | 10 (37.0) |  | 4 (21.1) | 10 (29.4) |  |
| Nursing home | 2 (7.7) | 0 (0.0) |  | 1 (5.3) | 1 (2.9) |  |
| Mech. Vent. (hours) | 115.5 [77.5 – 302.5] | 135.0 [94.0 – 305.0] | 0.682 | 116.0 [76.0 – 285.0] | 134.5 [90.0 – 329.0] | 0.522 |
| COPD | 1 (3.8) | 1 (3.7) | 0.978 | 0 (0.0) | 2 (5.9) | 0.281 |
| Pneumonia | 6 (23.1) | 12 (44.4) | 0.101 | 4 (21.1) | 14 (41.2) | 0.138 |
| SAPS II | 42.8 ± 10.9 | 53.1 ± 12.4 | **0.002*** ^(L 0.404)^ | 43.0 ± 12.2 | 50.9 ± 12.2 | **0.029*** ^(L 0.857)^ |
| APACHE IV^a^ | 68.0 ± 21.2 | 82.1 ± 23.0 | **0.028*** ^(L 0.935)^ | 67.6 ± 22.7 | 79.2 ± 22.5 | 0.087 ^(L 0.628)^ |
| I.V. antibiotics | 19 (73.1) | 25 (92.6) | 0.059 | 13 (68.4) | 31 (91.2) | **0.034*** |
| Cephalosporins (with or without other antibiotics) | 18 (69.2) | 23 (85.2) | 0.165 | 13 (68.4) | 28 (82.4) | 0.245 |
| Other antibiotics (with or without cephalosporins) | 4 (15.4) | 12 (44.4) | **0.021*** | 2 (10.5) | 14 (41.2) | **0.020*** |
| Only β-lactam antibiotics | 17 (65.4) | 17 (63.0) | 0.854 | 12 (63.2) | 22 (64.7) | 0.910 |
| Other antibiotics, with or without β-lactam antibiotics | 2 (7.7) | 8 (29.6) | **0.041*** | 1 (5.3) | 9 (26.5) | 0.058 |
| No antibiotics | 7 (26.9) | 2 (7.4) | 0.059 | 6 (31.6) | 3 (8.8) | **0.034*** |
| SDD topical antibiotics | 19 (73.1) | 23 (85.2) | 0.277 | 14 (73.7) | 28 (82.4) | 0.456 |
| Corticosteroids | 7 (26.9) | 9 (33.3) | 0.611 | 6 (31.6) | 10 (29.4) | 0.869 |
| Leukocytes |  |  |  |  |  |  |
| Sample^§b^ | 13.2 [9.3 – 18.7] | 11.4 [9.4 – 14.8] | 0.441 | 13.4 [9.0 – 19.5] | 11.5 [9.3 – 14.8] | 0.383 |
| Lowest^§§^ | 8.0 [6.3 – 9.3] | 7.9 [5.2 – 10.2] | 0.965 | 7.7 [6.0 – 9.4] | 8.1 [5.6 – 9.9] | 0.985 |
| Highest^§§§^ | 19.3 [15.4 – 22.4] | 18.2 [15.1 – 24.4] | 0.943 | 20.0 [14.3 – 22.2] | 18.1 [15.6 – 24.8] | 0.993 |
| CRP |  |  |  |  |  |  |
| Sample^§b^ | 66.5 [26.8 – 146.5] | 69.0 [41.0 – 122.5] | 0.920 | 68.0 [22.3 – 143.0] | 67.0 [43.1 – 125.3] | 0.825 |
| Lowest^§§^ | 3.6 [1.2 – 9.9] | 15.0 [3.3 – 54.0] | 0.057 | 2.9 [1.0 – 9.0] | 11.0 [3.1 – 56.8] | **0.017*** |
| Highest^§§§^ | 128.0 [79.0 – 217.5] | 133.0 [54.0 – 210.0] | 0.845 | 136.0 [82.0 – 240.0] | 130.5 [53.8 – 199.5] | 0.718 |

The average *S. pneumoniae* TIGR4 growth inhibition is based on one to four sputum samples, depending on how many sputum samples were obtained from each patient. ^§^Leukocytes/CRP measured in blood at the time of first sputum sample collection, ^§§^Lowest leukocytes/CRP measured in blood during ICU admission, ^§§§^Highest leukocytes/CRP measured in blood during ICU admission. Statistical analyses included the Pearson’s chi-squared test (n (%)), the Mann-Whitney U test (median [IQR]) and the t-test (mean ± standard deviation).

* p-values ≤0.05 are considered significant. ^L^ Levene’s test significance. ^a^Available for 50 patients; ^b^available for 49 patients.
